# E-Mental Health Interventions in Inpatient Care: Scoping Review

**DOI:** 10.2196/65947

**Published:** 2025-07-31

**Authors:** Alexander Diel, Isabel Carolin Schröter, Anita Robitzsch, Christoph Jansen, Martin Teufel, Alexander Bäuerle

**Affiliations:** 1 Department of Psychosomatic Medicine and Psychotherapy LVR University Hospital Essen University of Duisburg-Essen Essen Germany; 2 Center for Translational Neuro- and Behavioural Sciences University of Duisburg-Essen Essen Germany

**Keywords:** mHealth, inpatient, e-mental health, digital health application, acceptance

## Abstract

**Background:**

E-mental health (EMH) interventions are becoming an increasingly common topic of research, including in inpatient settings. These interventions provide accessibility and convenience, empowering individuals to take an active role in managing their mental health. By using personalized tools and resources, digital interventions can enhance the efficacy of delivering mental health care. Ultimately, they could offer a promising solution to the increasing need for mental health services.

**Objective:**

The objective of this scoping review is to provide an overview of the range, extent, and types of digital mental health interventions before, during, and after inpatient care in recent years, and to identify current gaps in the literature.

**Methods:**

The PubMed, Web of Science, and ScienceGov databases were searched. A second search was conducted in August 2024. The review included peer-reviewed studies published between January 1, 2015, and March 1, 2025, which were identified in 3 search rounds. The studies included adult patients receiving EMH-based care before, during, or after inpatient treatment. Only studies published in English or German and available to the authors were considered. Studies were assessed by 4 independent raters, and key information was summarized in shared documents.

**Results:**

Research on digital interventions in the context of inpatient mental health care has been increasing over the years. A total of 90 studies were identified. Some interventions were tested in multiple studies. Most (53/90, 59%) studies involved aftercare or interventions blending digital and face-to-face inpatient treatment. Twenty-six studies included a control group in their examination, and predominantly positive effects of digital inpatient and aftercare interventions were found. In general, positive acceptance was examined among patients and clinicians, which was assessed through questionnaires and interviews. Technical barriers and missing infrastructure were reported. Many studies included small sample sizes (23 studies had below 50 participants). Low adherence was a consistent limitation. Some aspects, such as inpatient preparation and EMH adherence interventions, showed promise, but detailed information was lacking. The majority of studies were carried out in Germany (n=50), followed by the United States (n=11).

**Conclusions:**

Research on the implementation of digital interventions before, during, or after inpatient care has been increasing in recent years, with initial promising results. Studies involving greater sample sizes and studies with more diverse patient groups need to be planned in the future. There are already indications that digital interventions can help maintain treatment benefits and somewhat improve symptoms in patients requiring inpatient treatment. The acceptance of EMH interventions was predominantly moderate to high, with structural issues stated as the most common barrier to use and acceptance.

## Introduction

### Background

Mental illnesses remain a major public and personal detriment to life satisfaction, physical health, mortality, and economics, yet only a portion of individuals with mental illnesses receive adequate treatment [1,2]. Internationally, the country income level is associated with adequate mental health treatment [3]. The same pattern is observed for low-income areas within a country [4,5]. One reason for this treatment gap is structural supply issues: people with mental health issues living in rural areas tend to lack adequate mental health care providers [6].

Mental health supply issues can be countered by digital tools providing mental health support, which have been summarized as e-mental health (EMH) programs. EMH programs are versatile. They can differ in their specific tools and technologies (videos, text messages, apps, websites, and virtual reality), the presence and intensity of human support (self-help, professional-guided, and peer-guided), their theoretical basis (eg, cognitive behavioral therapy [CBT]), their content (ie, specific therapeutic techniques used), and their application areas (eg, preparation, intervention, and aftercare) [7,8]. Furthermore, EMH programs offer several benefits over conventional mental health programs, such as increased outreach, screening, flexible monitoring, remote treatment, automatic functions, anonymity, and efficacy comparable to conventional treatments [9-12].

The effectiveness of EMH is well established for the outpatient setting [12-16]. This has been shown with a variety of meta-analyses regarding patients with different diagnoses [14,17,18]. However, it is unclear to what degree findings on EMH use in outpatient settings can be transferred to inpatient care. Inpatient care is indicated for cases that are too severe for outpatient treatment or that may require a combination of treatments [19], yet the use of EMH tools in outpatient care has mostly been investigated in mild to moderate cases. Inpatient care consists furthermore of a time-intensive treatment setting including multiple staff members, which differs from regular (eg, weekly or biweekly) meetings typical for outpatient settings. Hence, EMH tools may have to be adapted to fit the inpatient setting, which would require further validation.

However, research on EMH use in inpatient care is relatively sparse. Digital interventions can greatly benefit inpatient care and aftercare programs, for example, by additionally monitoring symptoms and risks of patients or enabling access to training and information before, during, or after inpatient treatment.

A recent meta-analysis found that inpatient EMH interventions (blended interventions and aftercare) show efficacy for a variety of mental health diagnoses [20]. Despite the promising synthesized results, the meta-analysis only included 26 randomized controlled trials (RCTs) focusing on efficacy, leaving out the results and perspectives of studies relying on other research designs or variables. Variables, such as acceptance and adherence, in the context of inpatient EMH interventions have not yet been synthesized or explored, and they are nevertheless important for the evaluation of EMH treatments [21-24].

Acceptance can be generally understood as the degree to which a treatment is considered appropriate by users and is seen as necessary for an intervention to be effective [25]. In the context of technology, *acceptance* can be defined as the use of a technological system resulting from a positive attitude towards it [26]. The Unified Theory of Acceptance and Use of Technology (UTAUT) considers *intention to use* to be indicative of acceptance [27]. The UTAUT may be used to measure acceptance a priori or without an intervention (ie, in the form of a survey), while other constructs related to acceptance, such as usability or satisfaction, are measured after the intervention and are thus associated with users’ actual experiences with EMH tools. Acceptance is of importance for EMH tools as high acceptance is associated with increased adherence [28], engagement [29], and eventually effectiveness [30]. Hence, acceptance is an important variable in the evaluation of EMH interventions. Given the variety of measures of acceptance in EMH research, this review will present acceptance outcomes as reported by the authors.

The discontinuation of treatment is considered a major problem in eHealth treatment [31]. In the context of EMH, *adherence* can be defined as the degree to which EMH tools are used as intended [32] and is related to *engagement*, which furthermore considers factors such as frequency, depth of use, and internal states of users [33]. Low adherence may distort the interpretation of outcomes, as cases with low outcomes may drop out. Yet despite its importance, an evaluation of acceptance and adherence is currently lacking. Since adherence is often measured in various ways [32], this scoping review will report adherence according to the authors’ terms.

Furthermore, the meta-analysis on EMH inpatient treatment [20] focused mainly on blended and aftercare settings, providing no information on other types of EMH implementations (eg, preparation, digital training, or remote treatment). Thus, other types of EMH implementations remain underexplored and will be focused on in this scoping review.

Systematic reviews or meta-analyses may not be sufficiently broad to present an exhaustive view (including outcomes beyond efficacy and different treatment settings) of the research. Hence, the goal of this scoping review lies in presenting a more holistic overview of the research field and applications of EMH tools in inpatient care beyond treatment efficacy for blended and aftercare interventions. For this reason, the scoping review will include a wide range of study designs (observational studies, protocols, essays and commentaries, reviews, and RCTs) without critical appraisal, as is typical for a scoping review [34].

Given the potential usefulness and acceptance of EMH tools, this review investigates recent findings of studies carried out before, during, or after inpatient care.

### Research Questions

The research questions have been defined as follows: (1) “What types of digital mental health procedures have been implemented and reported in inpatient care in recent years, and are those generally viewed as acceptable and effective?” and (2) “What are the current limitations and research gaps?” The aim of these research questions is to identify and summarize a wide range of research studies in the context of digital mental health in inpatient settings without specifying single measures, outcomes, or patient groups. Through this, a broad overview of the field is intended to be obtained.

## Methods

### Review

This scoping review follows the PRISMA-ScR (Preferred Reporting Items for Systematic Reviews and Meta-Analyses extension for Scoping Reviews) guidelines [34]. The research topic is explored to identify the main concepts and limitations in the current literature. A scoping review was chosen due to the relative novelty of the field and the heterogeneity of study designs, methods, and research topics, as well as due to the possibility of adapting the search term after the first systematic search. Relevant publications were classified in the categories of study protocol, EMH aftercare, online preparation before inpatient care, inpatient intervention, acceptance research, review, and others. The categories were decided a priori based on an initial assessment of the literature to identify common themes. The studies were further classified according to their EMH content based on the model proposed by Lin et al [8], which was chosen a priori. Their model consists of 5 main categories: area of application, intervention content, theoretical basis, human support, and technical realization. The model by Lin et al [8] offers a framework for categorizing and comprehending the wide range of digital mental health interventions available. This enables researchers, practitioners, and policy makers to evaluate, compare, and implement these interventions in clinical practice more effectively. No research protocol has been registered.

### Literature Search

The PubMed, Web of Science, and ScienceGov databases were searched for relevant literature. An initial search of PubMed was carried out to identify relevant articles and to develop an appropriate search term using text words contained in abstracts and index terms contained in the articles.

Secondary literature was identified by searching through the reference lists of relevant articles. After the search term was validated, a total of 3 searches were conducted. The second search used Medical Subject Headings (MeSH) terms for improved literature detection, while the third search included more recent publications.

To find all relevant studies, the terms “inpatient” and “ward patient” were used along with the term “digital” or “online.” Only studies with adult patients were included. The full search string used for PubMed was as follows: ‘(“digital”[tiab] OR “online”[tiab]) AND psychotherapy AND (“inpatient” OR “ward patient”) NOT children.’ The search string was used in the first search and designed to search for studies that included digital or online, psychotherapy, and inpatient or ward patient in the title or abstract. For the second and third searches, MeSH terms were used (*digital health*, *telemedicine*, *technology*, *mental health*, *mental disorders*, *psychotherapy*, *psychosomatic medicine*, *psychiatry*, and *inpatients*). Finally, the other databases were searched with the search string modified accordingly.

For the literature selection following the first search, every article of the original search was screened independently by 4 raters: ICS, AD, Patrick Wollenberg, and Lucy Powell. In the first screening, 3 independent raters (ICS, Patrick Wollenberg, and Lucy Powell) decided if an article was relevant by reading the abstract and full text, according to the inclusion criteria. In case of disagreements between the reviewers, a fourth independent rater was consulted until consensus was reached.

The second search was conducted on the PubMed and ScienceGov databases in August 2024 using MeSH terms. The results were screened, and eligibility was assessed by 2 independent raters (ICS and AD). In case of disagreements between the reviewers, they discussed the publications until a consensus was reached.

The third search was conducted in March 2025 using MeSH terms and an updated search string: (‘(“digital” OR “online” OR “mobile” OR “mHealth” OR “eHealth”) AND (“psychotherapy” OR “cognitive behavioral therapy” OR “psychodynamic therapy” OR “psychosomatic medicine” OR “psychiatry”) AND (“inpatient” OR “ward patient”) NOT children’). Results were screened by 2 independent raters. In case of disagreements between the reviewers, they discussed the papers until a consensus was reached. In the third search, 182 studies were found (71 in ScienceGov and 111 in PubMed).

Before consensus was reached, Cohen κ was at 0.78, indicating substantial agreement.

### Study Selection

First, duplicates were removed. Articles were then evaluated based on the following inclusion criteria by screening the title, abstract, and full text for relevant information within 1 stage by 3 (search 1) or 2 (searches 2 and 3) independent raters.

#### Peer-Reviewed Article

The article was published in a peer-reviewed journal.

#### Inpatient Setting

Given the focus on inpatient settings, articles were included if they focused on mental health in the context of an inpatient setting, including planned and released inpatients, as well as multiple settings if inpatients were also considered. Studies with an exclusive focus on outpatient settings were not included. Studies including both outpatient and inpatient settings were included, while reports on these studies focused on the results with inpatients.

#### EMH Tools

Given the focus on EMH tools, articles were included if they focused on EMH tools in the treatment of mental health. Digital procedures focusing solely on physical health or not designed to affect outcomes related to mental health (eg, online surveys on mental health with the purpose of collecting data) were not included.

#### Publication Year

Given the rapid technological development and improvement of EMH tools, older studies are expected to use outdated digital tools that may not represent current and future EMH technologies. More recent developments, innovations, and implementations in EMH include artificial intelligence (AI); virtual reality; chatbots; programs to detect emotion, voice, and cognition; various EMH apps; and general improvements in digital quality [35,36]. In addition, the COVID-19 pandemic further urged a transformation of EMH tools and services, leading to changes in both the quantity [37] and quality [38] of EMH tools. Hence, while earlier EMH literature focused on EMH tools as additives to treatment [39], contemporary literature focuses on a more holistic digitalization of mental health care [40]. Hence, earlier EMH literature may not adequately reflect the scope of contemporary EMH tools. As a cutoff date to exclude such earlier literature, articles were included only if they were published after January 1, 2015.

#### Language

Due to the authors’ lingual constraints, only articles in English and German were considered.

#### Availability

Finally, articles were included if they were available. Articles that could not be found in full length, even after an extensive search, were excluded.

### Data Charting

Data were charted according to the relevant variables identified in the research questions and included the following information: author names, article title, publication year, publication journal, country of data acquisition, type of intervention, key points, acceptance or critique of the EMH program mentioned in the article, mentioned study limitations, and recommended future directions. Data charting was performed using an Excel spreadsheet (Microsoft Corp) shared between all researchers, which is publicly available on Open Science Framework [41]. Data extraction was conducted by 1 rater and then verified by 2 independent raters.

The purpose of this scoping review is to provide an overview of EMH research in inpatient settings. Thus, the strengths and limitations of publications have been summarized without evaluation. After a broad overview of the selected literature, a narrative synthesis provides additional insights.

### Data Availability

Charted data and literature selection are publicly available on Open Science Framework [41].

## Results

### Overview

**Figure 1:**
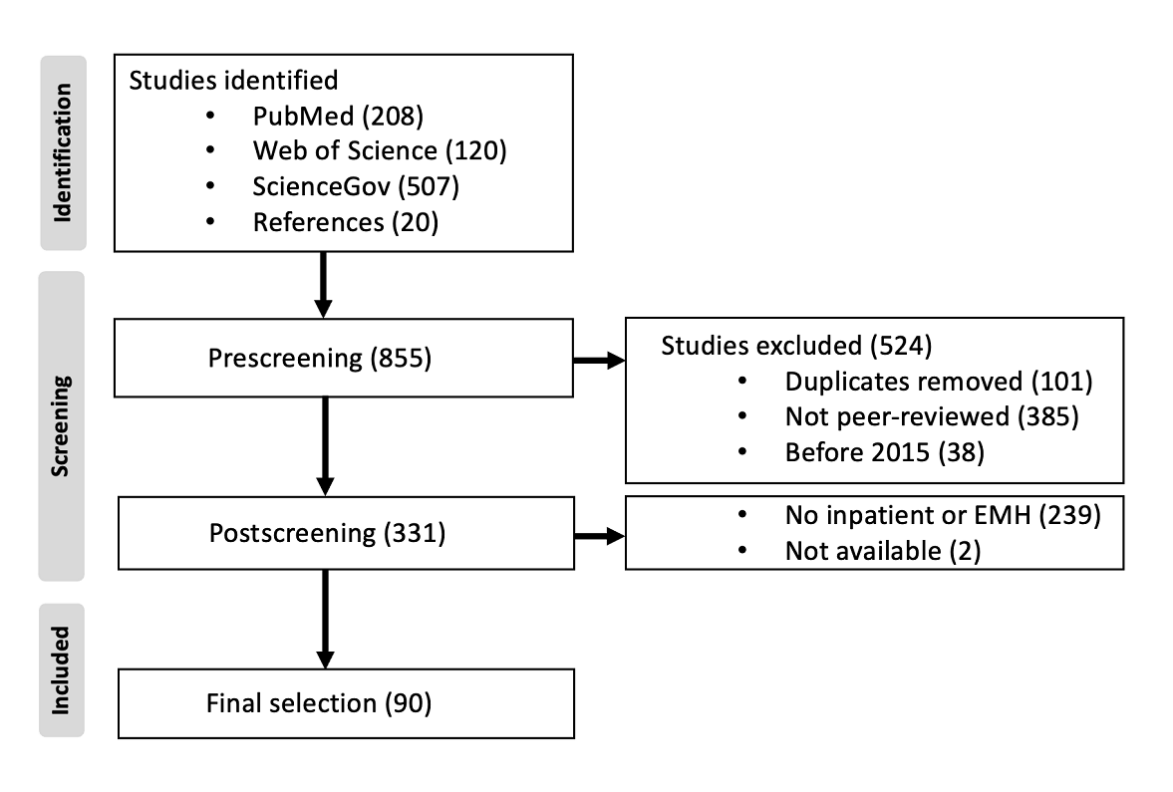
Flowchart of the study search and selection process. EMH: e-mental health.

Our research process identified 90 relevant publications in the 3 databases searched. A summary of the study selection is presented in Figure 1. We have provided the results for study selection following the first and second searches (Multimedia Appendix 1) and following the third search (Multimedia Appendix 2). In the third search, 24 additional studies were included.

Among the included studies, most were conducted in Germany (50/90, 56%). Moreover, 12 (13%) articles were reviews or comments, for which no specific country was recorded, as research from different countries was considered. Sample sizes varied across all included studies (15 to 11,237 participants), and various diagnoses were considered. The study distribution by country and year is summarized in Table 1.

**Table 1:** Distribution of the studies included in this scoping review by country and year.

Variable		Value (N=90), n (%)
**Country**		
	Germany	50 (56)
	United States	11 (12)
	Switzerland	6 (7)
	United Kingdom	4 (4)
	Canada	3 (3)
	Belgium	1 (1)
	Finland	1 (1)
	Iran	1 (1)
	Sweden	1 (1)
**Year**		
	2015	3 (3)
	2016	4 (4)
	2017	11 (12)
	2018	6 (7)
	2019	5 (5)
	2020	4 (4)
	2021	10 (11)
	2022	14 (16)
	2023	22 (24)
	2024	10 (11)
	2025	2 (2)

### Classification

Among the 90 articles, 45 involved clinical studies, of which 24 were RCTs. Among clinical studies, 30 focused on aftercare, 23 on blended inpatient treatment, 1 on preparation, 2 on station-equivalent treatments, and 1 on stepped care. Moreover, 16 studies focused on acceptance or qualitative assessments, 11 were reviews or meta-analyses, 9 were protocols, and 6 were essays or commentaries.

Information on aftercare and inpatient intervention studies is summarized in Multimedia Appendices 3 and 4.

#### Internet-Based Preparatory Treatment

Only 2 articles focused on online inpatient preparation. One study examined an internet-based preparatory treatment consisting of informational modules to prepare patients in advance of their inpatient stay and to reduce uncertainty about the treatment [42]. Results found significant improvements in psychological, physical, and social problems, although there was no difference in the length of hospital stay. Providers showed a high acceptance and saw numerous advantages such as sharing of clear up-to-date information, ease of use, quick accessibility, and low cost. Another study investigated patients’ and clinicians’ expectations of an online inpatient rehabilitation preparatory platform [43]. Patients mentioned text-heavy information as a limitation of contemporary online information that may be improved via visualization, videos, and adaptive information display. Preparation platforms may also be improved by focusing on the efficacy and long-term benefits of treatments.

#### Inpatient Intervention

Interventions included Deprexis (an online self-help program [44-47]), REMOTION (an emotion regulation tool [48,49]), Moodgym (a CBT self-help tool [50]), SlowMo (a cognitive restructuring tool for paranoid beliefs [51]), Minddistrict (a transdiagnostic self-help tool [52]), a digital documentation or information tool [53], interactive modules including trainings and information [54-58], a digital self-guided assessment [59], a group training to improve social skills [60], and CBT online training [61].

In general, studies on EMH-related blended inpatient interventions reported good acceptance [45,48,50,52,55-57,61] and high satisfaction [62]. Six studies also found a significant improvement in symptoms [44-47,54,55], while 1 study found no significant improvement [48]. No studies reported worsening of symptoms. Adherence was deemed low to moderate, with great variation across studies. The full completion rate ranged from 7.6% [54] to 93% [60]. Similarly, use ranged from 17% [57] to 95% [47]. A desired but low use of EMH tools has been reported in a survey on inpatient practitioners [63] and in a qualitative interview of EMH tools [64].

The studies including Deprexis (all implemented in Germany) reported significant reductions of depressive symptoms in the intervention group. Deprexis consists of digital modules in addition to inpatient treatment, with training, information, and questions for patients to answer at different follow-up times. Acceptance was regarded as good. According to the authors, refusal rates were low (30%) when compared to other studies containing an online intervention, which reported refusal rates as high as 57% [8,39,44].

One study examined the use of a digital monitoring tool called Tele-Board MED [53], which was implemented in addition to inpatient treatment to promote patient-provider collaboration in medical encounters. It was found to improve patient engagement in the treatment process, communication, and collaboration. However, acceptance was not reported, and no control group was assessed for comparison.

Two studies implemented programs to use and understand one’s own emotions during inpatient treatment through a digital application [54,55]. Both these studies included inpatient and outpatient groups, which showed significant improvements in anxiety [54], depression, and emotional competency [55]. Although both studies showed improved mental health and high acceptance, adherence was reported as relatively low, with only 7.6% [54] or 13.4% [55] of participants completing all units of the treatment plan.

Interactive modules were used in various ways in 3 studies [56-58]. The studies by Schwarz et al [57] and Van Assche et al [58] focused on patients with depressive symptoms, and the study by Hammond et al [56] considered substance use. The modules contained information as well as interactive elements in the form of training [57,58]. Among these 3 studies, only the study by Hammond et al [56] used a treatment as usual (TAU) control group, while no control group was assessed in the other 2 studies [56]. Two studies stated seemingly high self-reported acceptance, with a patient usage rate of 54% [57] or 58% [56].

In another study, online CBT was offered in addition to TAU for patients with anxiety [61]. Only overall satisfaction and acceptability were measured, and mixed results were reported. Although 86% of patients participated, the completion rate was rather low at 55%. Some participants found the application useful, but others mentioned negative aspects, such as the triggering of negative thoughts through the intervention, which were reported during semistructured interviews at the end. Anxiety and depression scores did not differ between those who received online CBT and those in the TAU control group [61].

Levis et al [59] examined a mindfulness intervention based on the Whole Health approach. The intervention offered diagnostic prompts to identify behavioral patterns and therapeutic exercises to change patterns. There was no direct question of acceptance, but different questions regarding satisfaction with the program were assessed, and participants showed high satisfaction with the intervention, according to the authors [59].

Wälchli et al [48] investigated the efficacy of using the REMOTION tool as an addition to inpatient psychiatric care. Although usability and satisfaction were good, adherence was low, and no significant differences in symptom severity were found.

Four studies conducted qualitative interviews with patients and staff on the use of blended EMH interventions [49,51,52,65]. The importance of human interaction and therapist guidance emerged in 2 studies, in addition to concern regarding technology and previous experience [49,51]. Two studies mentioned the fit of the implementation setting as a potential barrier [49,52], and 1 study focused on staff concerns such as increased workload and insufficient time [52]. One study mentioned concerns regarding patient safety and changes necessary for the inpatient unit [65]. Nevertheless, the qualitative interviews reinforced the use of EMH as blended complements, especially when combined with non-EMH social support, which was reported by patients [49,51,52].

In summary, the majority of studies reported promising efficacy, high acceptance, and good satisfaction of blended EMH inpatient interventions. The efficacy of blended EMH treatment was also found in a previous systematic review and meta-analysis [20]. However, adherence to EMH treatment was low, with wide ranges of adherence reported by studies. Studies generally remarked that further research must be carried out to confirm the results and to identify further aspects that should be considered.

#### EMH Aftercare

EMH aftercare is defined as online treatment starting after discharge from inpatient treatment.

Of the 90 articles, 13 were classified as EMH aftercare. The main goal of EMH aftercare was the outpatient maintenance or improvement of treatment gains achieved during inpatient treatment. EMH implementations included asynchronous mobile apps containing different modules [66-70], regular text messages with information regarding further treatment or information promoting mindfulness [71-73], chat forums with other former patients [74,75], online CBT [46,76-81], online self-management for anorexia nervosa [82-84], supportive-expressive therapy [85,86], ecological momentary assessment [87], and peer coach–based recovery [88]. Although EMH aftercare was mostly offered in relation to depressive symptoms, some treatment plans were focused on other diagnoses, including psychotic disorders and eating disorders. Control groups were used in all but 7 studies [74,81,83,84,86-88]. Some control groups received access to a selection of publicly accessible information regarding stress management and coping [68], mindfulness [71], depression [80], or health-promoting behaviors [85], or were waitlist control groups [73,77].

Overall, the results regarding depressive or affective symptoms were mixed. Five studies found significant improvements in depressive symptoms compared to the control group [46,55,68,73,77,80], while 1 study found none [67]. Significant symptom improvements were further observed in a transdiagnostic sample [77] and in work-related rehabilitation aftercare [85,86]. Meanwhile, 2 studies on eating disorders [77,82], 1 study on psychotic symptoms [72], and 3 studies on somatic comorbidities [70,75,78] found no significant improvements.

Acceptance was generally reported as high. Five studies reported good acceptance despite no direct measurement [55,67,68,71,77], and 8 further studies reported high acceptance with measures of acceptance, satisfaction, recommendation, helpfulness, or similar [69,72,75,77,79,81,83,86].

Reports of adherence were widely mixed, including assessments of low [73-78,82], moderate [68,70,71,85,86,88], and high [66,67,69,72,79,81] adherence. The rate of completion ranged from 1% [78] and 8% [74] to 95% [72] and 97% [67]. Similarly, the proportion of participants who used EMH tools at least once ranged from 36% [76] to 91% [85] or 100% [66].

Two studies conducted qualitative interviews on experiences with EMH tools (both involved ECHOMANTRA) [83,84]. Participants reported that despite general acceptance, increased personalization or tailoring may improve the experience. Social support, connections with other patients, and the involvement of carers were seen as great benefits. Self-monitoring reportedly increased participants’ self-awareness. The material was seen as useful and easy to access, yet the acceptability of remote support was mixed.

In summary, the results on the efficacy of EMH aftercare interventions were mixed to positive, complementing previous systematic reviews and meta-analyses on the positive effects of EMH aftercare [20,89]. Acceptance, satisfaction, and similar measures showed that EMH aftercare tools are generally well-received. However, aftercare studies reported wide variations in adherence and engagement, ranging from very low to very high. Thus, while EMH aftercare programs are promising and well-regarded tools for posttreatment inpatient stabilization, low and unpredictable adherence remains a limitation for reliable implementation.

#### Remote Treatment

Two studies focused on remote treatment procedures, with EMH interventions fully replacing indicated inpatient treatment [90-92]. Rauschenberg et al [90] described inpatient-equivalent treatment as an alternative that fulfills the requirements of psychiatric inpatient treatment conducted in a home environment. EMH tools, including ecological momentary assessments and digital feedback, further allow the implementation of adaptive digital interventions that adapt to the daily needs and momentary assessments of patients. Farrington et al [92] meanwhile conducted qualitative interviews and remote treatment as alternatives to inpatient treatment implemented when the health care system was disrupted during the COVID-19 pandemic: remote home care was found to be a satisfactory alternative to inpatient care, although the importance of social connections was emphasized.

In general, research on EMH-supported inpatient treatment alternatives is lacking, yet limited results are initially promising.

#### Acceptance and Qualitative Interviews

As previously mentioned, the acceptance and satisfaction of blended inpatient treatment or aftercare were generally good. Beyond these, acceptance was examined as the primary focus in several studies. These studies were conducted in a variety of settings, including inpatient [24,64,89,93-97] and inpatient-outpatient mixed [98] facilities. Both patients and professionals were assessed, and the results were reported in several studies. Results regarding patients were either obtained through specific acceptance questionnaires, including the UTAUT [89,93], a patient preference survey [98], USE [95], and semistructured interviews [96]. The acceptance of interventions from the perspective of health professionals was also evaluated, with questionnaires based on the UTAUT model [89,94,97]. The professional perspective was predominantly evaluated through the analysis of individual responses, which were obtained through conducting interviews by sending out a qualitative survey [24,95], through semistructured interviews [99], or with a direct questionnaire [51] by solely assessing user experience. Greenwood et al [51] demonstrated a beneficial outcome by assessing core themes related to the approaches and challenges of technology or improvements in paranoia and well-being. Another study [100] interviewed health care professionals regarding the use of inpatient telepsychiatry: telepsychiatry generally improved the care process by addressing the needs of patients and carers. Dewa et al [101] found passive monitoring systems in forensic psychiatric hospitals to be generally acceptable, although acceptance was decreased when knowledge of the technology was low. Hennemann et al [94] offered a review on current studies highlighting acceptance among health care professionals. In general, 88% of participants among all studies reported low to moderate acceptance, with only 21% of all participants indicating a sufficient eHealth training. They did find, however, that 75% would reconsider using an eHealth intervention after appropriate education.

The majority of studies found moderate to high acceptance either through direct measurement with validated tests or through an analysis of qualitative answers [45,48,50,52,55-57,61,69,72,75,77,79,81,83,86]. However, 3 studies [89,93,97] reported low or mixed acceptance, such as low to moderate acceptance for general acceptance, as well as moderate acceptance among patients (57.1%) but low acceptance among professionals (29.6%) [89]. Sander et al [97] demonstrated a low acceptance rate, as measured by the Attitude toward Telemedicine in Psychiatry and Psychotherapy scale, with mixed responses for specific questions such as agreement to online therapy as a good addition (77%) or recommendation of online therapy to patients (36%). Some studies reported that patients hold a predominantly positive attitude toward online therapy [64,95,98], although 1 study also highlighted a high dropout rate of 43% [95]. Differences in acceptance rates between studies may be due to different methods of assessing acceptance and differences in knowledge or technical prerequisites.

In summary, the acceptance of EMH tools is generally high and tends to be higher for patients than for health care staff. Acceptance may be further improved by (1) increasing transparency and knowledge regarding EMH tools, including staff training [98,101]; (2) improving aspects of EMH content, such as visualization [43] and personalized tailoring [83]; (3) facilitating the role of social support, social connections, and caretaker guidance [49,51,83,92]; and (4) considering staff’s workload and time constraints [52,62]. However, high dropout rates may bias the results on high acceptance as low acceptance by patients may be a reason to stop treatment.

#### Improvement of Digital Acceptance and Digital Literacy

As previously mentioned, adherence to EMH-supported interventions varied greatly across studies and tended to be low. This is despite the promising efficacy and good acceptance of EMH tools. Two studies reported good usability but low actual usage [63,64], despite the majority of patients in inpatient care having access to mobile devices [100]. Hence, adherence appears to be a major limitation in the realization of EMH-supported interventions.

Two studies implemented interventions to improve digital acceptance and literacy [55,102]. Kreis et al [55] implemented an acceptance intervention for the online self-help tool KEN-Online, which increased acceptance and initial use by 20% compared to the passive control group, yet adherence as measured by the rate of completers remained low at 13.4%. Hence, the acceptance intervention mainly showed initial effects. Camacho et al [102] conducted DOORS digital literacy training. Although digital skills were improved by the intervention, there were no significant effects on symptom reduction, and adherence was not reported.

Given the limited number of studies and modest findings, an empirical investigation on interventions to increase EMH tool adherence remains an open issue.

#### Reviews

Eleven articles included in this scoping review are reviews or meta-analyses [20,89,103-111]. Among these, 1 systematic review by Henneman et al [89] focused on the efficacy of EMH inpatient aftercare based on 16 RCTs, and 1 meta-analysis by Diel et al [20] focused on the efficacy of blended and aftercare EMH inpatient treatment based on 26 RCTs. The results support the efficacy of EMH inpatient treatment, concerning both blended treatment and aftercare, and across a variety of mental disorders. However, both reviews noted the limitation of the small number of studies, especially for subgroup analyses. Hennemann et al [89] further noted partial inconsistencies in results for EMH aftercare treatments.

Three reviews focused on EMH-supported interventions for both inpatient and outpatient treatment [103,104,110]. Erbe et al [103] found that blended treatment may improve time efficiency and the dropout rate, yet they remarked about a lack of appropriate research. Dülsen et al [104] noted the initial promise of EMH tools in inpatient treatment but called for further research on potential barriers and the development of proper protocols. Ehrt-Schäfer et al [110] also found promise for blended psychotherapy yet cautioned against generalizations.

The remaining 6 reviews focused on the treatment of specific disorders or symptoms, including but not necessarily limited to inpatient and EMH interventions [105-109,111]. Sweetman et al [105] investigated CBT treatment for insomnia on sedative-hypnotic use and found promising yet limited results from digital CBT. Spanakis et al [106] investigated behavioral smoking cessation treatment but found limited results for digital treatments. Liu et al [107] found that online mindfulness-based interventions can elevate symptoms in patients with physical health symptoms. Castle et al [108] found an initial promise of digital interventions for obsessive-compulsive disorder. Loh et al [109] investigated mobile health interventions in the treatment of psychotic disorders, found a wide variety of applications and settings, and urged holistic evaluations of EMH applications. Finally, Shin et al [111] investigated the trends of EMH applications for suicide prevention. The interest in digital suicide prevention is increasing and includes postdischarge follow-up, screening or assessment, and safety planning, but research is lacking, especially on long-term outcomes.

#### Protocols and Essays/Commentaries

Nine of the included articles were study protocols [112-120], and 6 studies were essays or commentaries [90,121-125]. Baumeister et al [121] provided a description of blended psychotherapies, dividing them into sequential blended treatments, including stepping up (digital interventions preceding in-person treatment, for example, as a preparation or to bridge waiting times) and stepping down (digital interventions following in-person treatment, for example, as aftercare), and integrated treatments, including complementing digital sessions, resource-driven replacement of in-person sessions, and improvement of in-person sessions using digital tools. Rauschenberg et al [90] presented inpatient-equivalent treatment using digital support. Hirjak et al [122] emphasized the ethical and scientific challenges of implementing EMH in mental health treatment. Feinstein [123] presented and suggested the use of EMH tools in crisis intervention psychotherapy during the COVID-19 pandemic. Inchausti et al [124] commented on the challenges caused by the COVID-19 pandemic and the potential benefits of telepsychiatry regarding those challenges, including underestimation of damage, resource deficits, poor planning and coordination, and risks of early crisis responses. Finally, Westheimer et al [125] presented a model to implement digital tools in inpatient mental health care, the Technology Implementation for Mental Health End Users (TIME) framework, focusing on the interplay and communication between “high-tech” digital tool development and “high-touch” in-person treatment.

#### COVID-19–Related Studies

Five articles focused on the use of EMH during the COVID-19 pandemic [91,123,124,126,127]. One study examined the experiences of patients during the COVID-19 pandemic [126]. One article was a commentary on telepsychotherapy during the pandemic [124]. Another article provided a description of crisis intervention during the pandemic [123], and another article mentioned the effect of online psychoeducation on patients during the pandemic [127].

Favorable views have been reported by Schlegl et al [126], Inchausti et al [124], and Shaygan et al [127], with the latter reporting successful implementation with the mitigation of stress and promotion of resilience. These articles did not provide a conclusive overview since they were very specific in their research questions and therefore could not be compared [124,126,127].

#### Others

Four articles were classified as “others” [43,62,63,128]. The articles presented an overview of the perspectives of rehabilitants regarding digital information before inpatient psychosomatic care [43], a description of eHealth use in inpatient mental health units regarding barriers or benefits [63], a description of digital symptom monitoring offered during inpatient treatment [62], and a survey on EMH inpatient use [128]. The articles reported EMH tools as potentially helpful according to participants, and the article on the information program before inpatient care reported uncertainty regarding effectiveness but outlined the importance of familiarizing patients with the effectiveness and sustainability of the inpatient program [43]. Chat- and phone-based EMH tools were most commonly used according to a survey [102]. One study showed that patients preferred the digital version of symptom monitoring to the analog version [62]. The main barriers reported in the survey filled out by staff in inpatient units were unclear roles, dwindling implementation support, a high workload, and a high number of administrative tasks [63].

#### Summary: EMH Tools Along the Inpatient Journey

**Figure 2:**
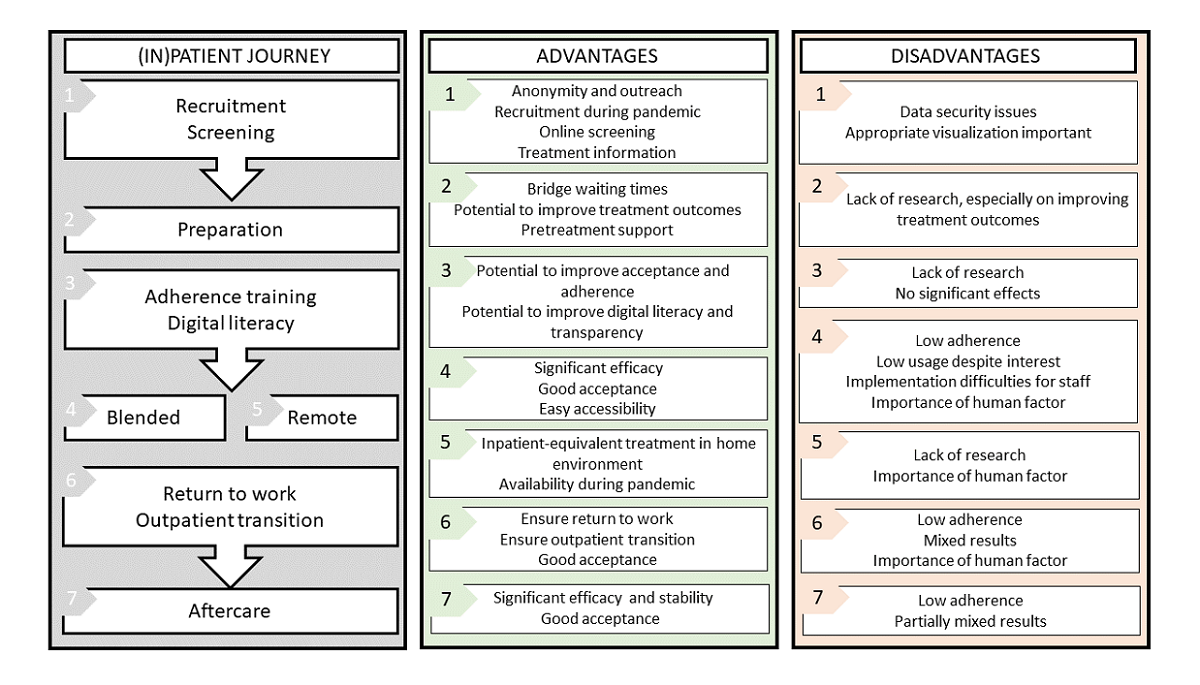
Potentials and concerns of e-mental health tools along the inpatient journey.

A summary of the use of EMH tools and their potentials and concerns based on the scoping review is shown in Figure 2.

The inpatient journey can be divided into 3 treatment steps (preparation, inpatient treatment, and aftercare) and 3 transitory steps (recruitment/screening, adherence/digital training, and return to work/outpatient transition). Along all 6 steps, the implementation of EMH tools has been investigated in research. Across treatment steps (preparation, blended/remote treatment, and aftercare), both efficacy and acceptance have been shown [20]. However, low adherence has remained a consistent issue for the implementation of EMH tools [63,64]. Although EMH-assisted inpatient preparation shows potential [42], research is currently lacking. Digitally assisted recruitment and screening can allow a wider outreach of patients and efficient provision of information [9,11], and interventions focusing on the improvement of digital treatment (eg, adherence and digital literacy training) may counteract the common disadvantages of EMH tools, most notably low adherence. However, such interventions also show limited research, which, thus far, has not shown promising results [55]. Although posttreatment return-to-work treatments show promise [68], other types of posttreatment transition-based interventions have shown limited effectiveness [60,82].

In summary, while research generally supports EMH-assisted treatment in terms of efficacy and acceptance, low adherence remains a common issue. While transitory and preparatory interventions with EMH support are promising tools to enhance the inpatient journey, there is currently limited research available for their critical evaluation.

## Discussion

### Overview

EMH tools in inpatient settings are gaining increasing importance and use. The aim of this scoping review is to present an overview of the current research on EMH tools in inpatient settings and to identify core research themes and gaps. EMH tools are primarily used in blended and aftercare inpatient settings, with some research investigating their use in preparation, digital training, and remote treatment. Research generally shows promise regarding the use of EMH tools in inpatient treatment, with high acceptance and efficacy. However, low adherence remains a prevalent issue.

EMH inpatient treatment is a versatile field used for various diagnoses and involves a wide range of tools and different treatment settings or stages (Figure 2). Despite promising results in some parts of implementation (especially blended and aftercare treatment), several potential areas remain understudied.

### Types of EMH Interventions

Mobile apps, text messaging, and web-based intervention programs containing different kinds of content modules were assessed. Digital aftercare treatments were frequently implemented, and their results were researched the most (17 of 65 studies) [66-80,85,86], although digital inpatient interventions have been increasingly researched in recent years (13 of 65 studies) [44-47,53-61]. Internet-based preparatory treatment was only implemented and assessed in 1 study [42]. Many of the included studies featured aftercare to maintain treatment effects after inpatient treatment or to improve symptoms in patients through guided or unguided tools. Inpatient programs are aimed to be used in combination with in-person inpatient treatment sessions, as well as sometimes continuing into aftercare treatment. Additionally, programs have been implemented primarily as an online equivalent of inpatient care, and this was assessed in 2 studies. Although only 1 of the 2 studies evaluated effectiveness and the other focused on the monetary benefit, promising results were found in favor of digital mental health treatment in the form of video-based therapy. This must be assessed further, as a single study does not allow for generalization, even with a large sample size and the use of a control group with in-person inpatient treatment.

Digital treatment was facilitated by both the use of mobile phones and the use of computers. In our review, more studies featuring mobile phones were used for delivering digital mental health treatment than studies featuring computers. This corresponds to the results of a scoping review by Petrovic et al [129], which also found more studies assessing the use of mobile phone apps. This may be attributed to the easier accessibility of mobile phones and the prevalence of mobile phone–reliant programs [100,130].

Assessed treatment programs were based on a variety of theories, including CBT, mindfulness, emotion regulation training, supportive-expressive therapy, and peer-recovery coaching. CBT is based on the theory of learning and cognition and presumes that psychological problems are related to maladaptive ways of thinking and behaving. Hence, CBT aims to improve mental health by shifting maladaptive ways toward adaptive ways of thinking and behaving. EMH-based CBT has used tools such as thought and behavior diaries, behavior observation, daily structure, cognitive restructuring, solution-oriented thinking, and psychoeducation [44-47,50,51,57,59,61,67,76,78,79,81,131]. Mindfulness interventions presume that through practices aimed at increasing awareness of the present moment (eg, mindful meditation), mental health can be improved by reducing stress and worries via awareness and nonjudgmental interpretations of thoughts and impulses. As mindfulness interventions require continuous exercises, EMH-based mindfulness interventions typically aim at providing users with such exercises in a flexible manner [54,59,71]. EMH-based emotion regulation training presumes that mental health can be improved via the identification, selection, and implementation of emotional states [48,49]. Supportive-expressive therapy was implemented by allowing patients to express themselves via blog posts, which were commented on by a therapist [85]. Peer-recovery coaching was performed via daily contact and support with a coach who recovered from substance abuse.

Some EMH-based interventions combined tools from different schools, such as Deprexis; hence, overlap among these treatments was noted.

### Treatment Outcomes

Consistent with previous systematic reviews and meta-analyses [20,89], the majority of studies (n=21) in this review found positive and significant results of EMH-based treatment, especially for blended and aftercare settings. However, not all studies included control groups, limiting interpretations on efficacy.

### Acceptance

In general, EMH programs have a moderate to high acceptance among patients and experts, according to most studies (n=27). Acceptance was measured as the primary focus in 11 studies, but it was also assessed in other studies using either self-conceptualized questionnaires or validated questionnaires, which primarily assessed the effectiveness of a specific program. To recruit patients, the EMH programs were offered to inpatients either for an additional digital intervention or as an aftercare program after the inpatient stay. Therefore, only interested patients were assessed in the acceptance studies, which could skew the results toward a higher acceptance.

Interestingly, it does not seem that acceptance interacts with treatment outcomes. Acceptance was generally reported as high across the majority of studies (n=27), including studies that did not show a significant symptom reduction [48,72,75]. This, however, may be due to the various ways through which acceptance was measured and reported in the studies; hence, comparisons between studies are difficult.

### Identified Limitations and Concerns

The scoping review identified several limitations and concerns that occurred throughout the research.

#### Low Adherence

In 8 studies investigating adherence, dropout rates were higher in interventions containing some sort of online element than in face-to-face interventions due to low adherence. This could have impacted the measured acceptance or effectiveness as low acceptance of effectiveness cases may have dropped out [72,94]. Additionally, willingness to participate was generally low, with various authors describing a low percentage of patients interested in participating. As a result, sample sizes were sometimes not achieved, and unwilling patients were assessed as a control group. This could have affected the treatment effects. Although adherence-improving interventions have been investigated, their results remain modest [55].

#### Methodological Quality

Given the wide range of included studies, several concerns on methodological quality emerged. Future research should increase sample sizes as many presented samples were small, undermining statistical power. Replications featuring larger sample sizes are necessary to ensure representativeness and generalizability.

The included studies additionally predominantly featured specific patient groups, sometimes without a control group. For future research, programs should be tested in comparison with a control group and relevant patient groups to investigate significant effects and assess efficacy.

Acceptance measures were heterogeneous, and many studies did not directly measure acceptance in any way. Future research should aim to use standardized measures of acceptance to allow proper assessment and comparisons between studies.

#### Data Security Concerns

Concerns about data security and privacy are common in the context of EMH [11], which is especially relevant given the vulnerability of psychiatric patients. Patients may experience an invasion of privacy caused by passive monitoring systems and an imbalance in the power dynamic [101] or by sensitive data being shared [124]. Clinical staff also noted data security risks caused by digital data storage, for example, through hacking attempts [99]. In 1 survey, privacy concerns were reported by 35.9% of practitioners [24]. Many data security concerns on EMH tools in general can be applied to inpatient settings, such as risks caused by digital security breaches associated with hackers or corporations [132,133]. Inpatient settings allow new data security risks to emerge, for example, through breaches caused by other inpatients [124]. However, the presented literature has not sufficiently presented solutions to potential security concerns. Future research may focus on security concerns in the context of EMH inpatient care.

#### Role of Human Support

The concern of EMH tools substituting human interaction has been raised in the past [134]. In inpatient settings, qualitative interviews showed that the importance of human interactions has been raised by both patients and clinical practitioners [24,49,51,83,92], and EMH tools in inpatient care showed increased acceptance when framed as enhancements rather than replacements of face-to-face treatment [99]. Meanwhile, EMH tools showed the greatest effectiveness when used and supported by humans [135]. In order to minimize risks and fears about EMH tools replacing face-to-face human interaction, practitioners may focus on implementing EMH tools alongside human interaction if possible and enlighten patients on how EMH tools are supposed to enhance, rather than replace, in-person treatment.

#### Limitations in Implementation

Practical concerns on the implementation of EMH tools are prevalent in inpatient settings. Those include concerns on the fit of EMH tools in inpatient settings [49,52], insufficient digital infrastructure [63], staff workload issues [52,62], and lack of knowledge or expertise among staff or patients [24,49,63,101]. The variety of implementation limitations suggests the need for proper preparation of the inpatient setting, infrastructure, treatment, and clinical staff in order to efficiently implement EMH tools into inpatient treatment routines.

### Identified Research Gaps

Several research gaps were identified through the scoping review.

#### EMH Inpatient Preparation and Digital Literacy or Adherence

There is a lack of research on both EMH-supported inpatient preparation and interventions aimed at improving EMH-supported treatment. This is surprising given that both aspects show noteworthy potential: EMH-supported inpatient preparation may be used to improve inpatient treatment efficacy while also providing an opportunity to support patients during the vulnerable waiting time. Meanwhile, interventions aimed at improving EMH-supported treatment (eg, digital literacy or digital adherence training) may counter common limitations and concerns of EMH treatment, namely lack of knowledge or experience and low adherence. An EMH-based preparation may even combine inpatient preparation, waiting time bridging, and digital training to support and prepare patients for an EMH-supported inpatient treatment. Hence, such preparatory interventions may be investigated in future research.

#### Diversity in Patient Demographics

The role of demographic and clinical diversity in the acceptance and efficacy of EMH-based treatment has not been sufficiently explored. Subgroup analyses based on patient diagnoses showed EMH efficacy for various disorders [20]; however, the number of studies was too small to conduct proper subgroup analyses. One study did not find demographic moderators for symptom outcomes [47]. On the other hand, EMH tools may be tailored to specific demographics to increase efficacy or acceptance [64]. Understanding the interactions between patient demographics and EMH tool acceptance and efficacy may help by providing specific recommendations and allowing tailoring of EMH tools to patients’ needs according to their demographics.

#### EMH-Assisted Remote Treatment

Remote alternatives to inpatient treatment may also be further investigated. Inpatient-equivalent treatment (German: stationsäquivalente Behandlung [StäB]) has been established in Germany recently and can be used in medically appropriate cases, in which the treatment can be allocated. It was established in 2017 and sets the same requirements regarding patients as for inpatient treatment in a ward. Patients are offered the possibility of one-to-one meetings with a therapist while in treatment as well as group therapy, and accompaniment and training in daily life. As a requirement, all people living in the household must agree to the treatment, although an opportunity to talk with the patient privately must be given. Daily contact must be possible between patients and treatment providers, and documentation must be to the same standard as inpatient treatment. Not all clinics offer the inpatient equivalent for outpatient care so far, but it is being implemented more frequently [136]. Considering the growing evidence of EMH, this concept might be extended by adding EMH to the treatment plan.

#### Long-Term Stability

Although a prior meta-analysis showed long-term stability [20], the majority of research on EMH tools did not investigate long-term sustained improvements via follow-ups. Future research may focus on the long-term benefits of EMH-based inpatient interventions.

### Limitations of This Work

There are limitations in this work. Publication bias may lead to studies with nonsignificant findings not being published, skewing the results of this scoping review toward positive outcomes; however, a meta-analysis on the efficacy of EMH tools in inpatient settings could not find evidence for publication bias in this field [20]. The range of studies in this scoping review was higher compared to the meta-analysis, allowing potential publication bias to emerge. Furthermore, the number of included studies may have been limited because data were not always fully reported. Additionally, it should be noted that only the bibliographies from the included studies were reviewed, which means that there is a possibility that some studies cited in the articles reviewed and excluded were missed.

Given the nature of a scoping review, a risk-of-bias assessment was not conducted; hence, the quality of the included research studies was not taken into consideration. A wide range of study designs was included in this scoping review (eg, RCTs, observational studies, essays, and reviews). Summaries of reported results should be interpreted as trends in the research field rather than research results. General methodological limitations among the studies included small sample sizes, low adherence, no randomization, and lack of a control group. The limitations and barriers in conducting online studies may have contributed to the ambiguous results.

Study selection can be distorted by spectrum bias and random error [137]. Spectrum bias may have occurred due to sample restrictions, such as including nonadult patients and samples focusing on neurological or neuropsychological conditions. Hence, the results of this scoping review may not be generalizable to these samples. Furthermore, only literature in English and German was selected, risking bias by not including potential literature in other languages. Random errors may stem from errors in the selection of literature and the charting of data; however, the inclusion of multiple independent raters can reduce this error.

Notably, a high number of studies have been conducted in Germany. A reason might be that mental health inpatient treatment is more frequent in Germany than in other countries. Hence, studies using these programs in other languages and with other cultural groups should be assessed in the coming years.

Published literature experienced a large increase in the years following the COVID-19 pandemic, with a peak in 2023. This trend may not only reflect the general increasing interest and improvement of digital tools but also the recognized importance and potential of digital tools in times when the regular health care system is disturbed. Furthermore, the clinical use of AI tools has emerged in recent years. Although this review did not identify any research using AI-based EMH tools in inpatient settings, such tools may become the topic of future research. AI-based tools may come with unique issues and concerns, such as privacy concerns and unsupervised responses by AI chatbots [138,139]. As AI-based tools find implementation in inpatient settings, AI-specific challenges have to be taken into consideration.

### Conclusion

EMH treatments have the potential to significantly improve patients’ symptoms through aftercare to extend inpatient treatment benefits as well as support therapy during inpatient care. This research field has been growing in recent years, although more studies, including longer programs and comparisons with control groups, are needed. A variety of patients with diverse diagnoses have reported improvements in their symptoms when using a range of digital treatment options. Low adherence remains a recurring limitation. Some potential areas, such as EMH-based preparation and adherence improvement, are not yet well researched.

## Appendix

Multimedia Appendix 1 Flowchart of the study search and selection process for the first 2 searches.

Multimedia Appendix 2 Flowchart of the study search and selection process for the third search.

Multimedia Appendix 3 Summary of studies focusing on blended inpatient interventions including e-mental health.

Multimedia Appendix 4 Summary of e-mental health aftercare studies included in this scoping review.

Multimedia Appendix 5 PRISMA-ScR (Preferred Reporting Items for Systematic Reviews and Meta-Analyses extension for Scoping Reviews) checklist.

## Abbreviations

AI: artificial intelligence
CBT: cognitive behavioral therapy
EMH: e-mental health
MeSH: Medical Subject Headings
RCT: randomized controlled trial
TAU: treatment as usual
UTAUT: Unified Theory of Acceptance and Use of Technology
