# Supplementary figures and images for: E-Mental Health Interventions in Inpatient Care: Scoping Review
Source: J Med Internet Res. 2025 Jul 31;27:e65947. doi: 10.2196/65947 (PMC12355140; doi:10.2196/65947)

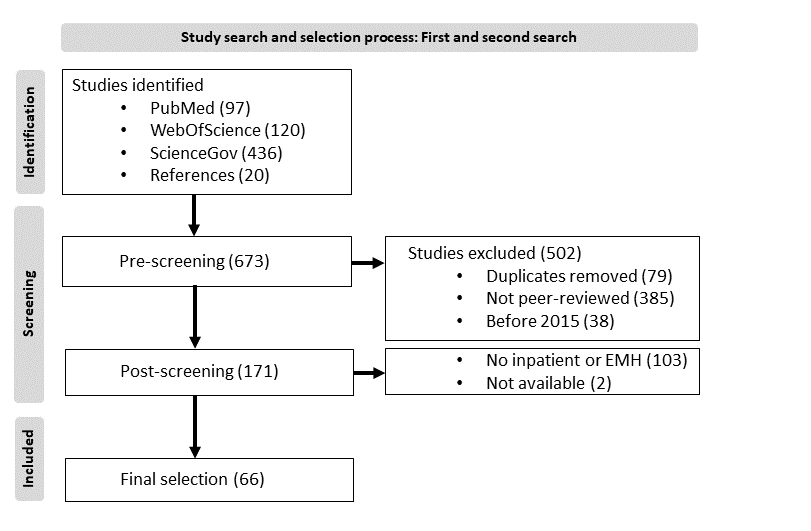

Supplement: Multimedia Appendix 1 [file jmir_v27i1e65947_app1.png]

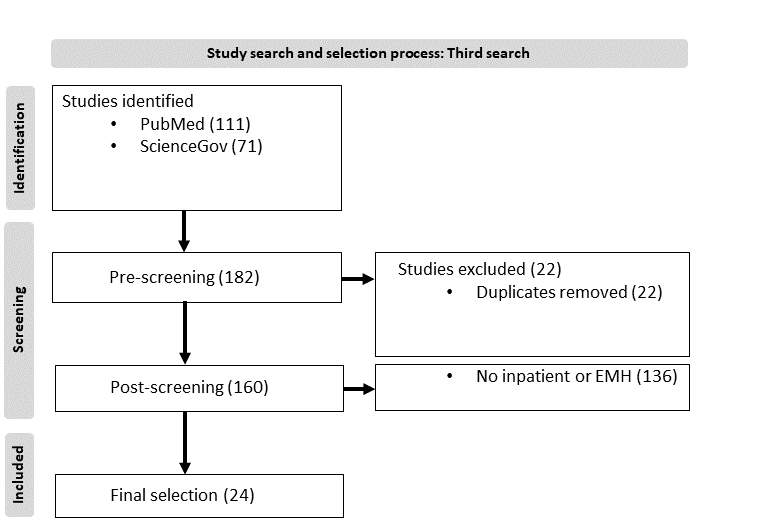

Supplement: Multimedia Appendix 2 [file jmir_v27i1e65947_app2.png]
