# Supplementary material for: E-Mental Health Interventions in Inpatient Care: Scoping Review
Source: J Med Internet Res. 2025 Jul 31;27:e65947. doi: 10.2196/65947 (PMC12355140; doi:10.2196/65947)
Supplement: Multimedia Appendix 3 [file jmir_v27i1e65947_app3.docx]

**Multimedia Appendix 3.** Summary of studies focusing on blended inpatient interventions including e-mental health.

| **Author (year)** | **Patient** | **Control group** | **EMH type** | **Theoretical background** | **Content** | **Results and Acceptance** | **Adherence** |
| --- | --- | --- | --- | --- | --- | --- | --- |
| Perlich & Meinel [1] | Inpatient, different diagnoses | no control group | Documentation tool (Tele-Board MED) | Patient empowerment | Support creative teamwork, documentation tool with active involvement of the patient | increased patient engagement and improved collaboration, communication and integration in consultations | No patient stopped |
| Zwerenz et al. [2] | Inpatient, depressive symptoms | TAU (weekly online information on depression) | Deprexis | CBT, positive psychology, emotion-focused therapy, and dream work | Self-help tool with 10 modules | depressive symptoms significantly lower in intervention group, also anxiety, quality of life and self-esteem | Almost half of discharged patients used Deprexis |
| Zwerenz et al. [3] | Inpatient, depressive symptoms | TAU (weekly online information on depression) | Deprexis | based on the self-help book Living Like You Mean It by Ronald J Frederick | Self-help tool with 10 modules | Increased emotional competence of patients with mental disorders. self-report: 52% program as good and 26% as very good --> 78% total | 73.9% completed assessment at end of intervention |
| Hammond et al. [4] | Inpatient, substance abuse | TAU (not specified) | web-based Therapeutic Education System (TES) | modules on substance use-related topics | program consistent of 65 interactive modules (effective problem solving, HIV and AIDS, drug refusal skills training) in text and/or video | High acceptability of modules | less than 1% of patients declined to participate --> suggests general interest, high ratings of acceptability were found |
| Bendig et al. [5] | Inpatient, different diagnoses | No control group | Online modules | evidence-based group training of social skills | complementary online modules additionally to face-to-face therapy | Participants were satisfied with the blended SST concept as a whole.  Patients acceptance towards digital therapy slightly decreased from T0 – T1 (non-significant), general Rating of online modules: 8/10 | 93% completed pre- and post-measurements |
| Nolte et al. [6] | Inpatient and outpatient, depressive symptoms | TAU (not waitlist) | Deprexis | CBT, positive psychology, emotion-focused therapy, and dream work | Self-help tool with 10 modules | Significant improvement effects in intervention group found, acceptance not assessed | 95.28% used intervention |
| Kreis et al. [7] | Inpatient and outpatient, different diagnoses | TAU (former patient group) | Informative lecture to boost acceptance of KEN-Online | Acceptance facilitating intervention, TAM theory | Acceptance facilitating informative meeting to increase acceptance of transdiagnostic psychodynamic self-help program with modules | Acceptance nearly 20% higher in intervention group than control group | 13,4% completers |
| Sharma et al. [8] | Inpatient, anxiety | TAU (inpatient care) | App for anxiety symptoms (MindShift CBT) | CBT | App containing CBT-based tools, information about anxiety, meditation exercises and defining of personal goals | Overall positive scores on CSQ-8 questionnaire (mean 20.2, possible range 8-32) | Scores on user-experience questionnaire (mean 80.5, possible range 0-110) reflect moderate to high engagement |
| Schwarz et al. [9] | Inpatient, depression | No control group | Online self-management tool against depression | CBT | Six modules regarding different topics | 67% of participants wanted to continue using the tool post inpatient setting | 17% of participants used tool at least once a week |
| Van Assche et al. [10] | Inpatient and outpatient, depressive symptoms | No control group | Moodbuster, modular web-based platform with connected smartphone application for monitoring | CBT | Modules regarding psychoeducation, activities, problem solving and positive thinking | Client satisfaction with CSQ-3 measured mean 5.56 out of 12 | 33.3% completed post-survey, no other data for adherence |
| Levis et al. [11] | Inpatient, substance abuse | Mindfulness-based stress reduction of UC San Diego | Conflict Analysis, a self-guided intervention | Whole Health | Diagnostic prompts to identify behavioural patterns and therapeutic exercises to change patterns | High agreement with questions regarding the intervention | 73.3% of participants completed all units in general (not only digital intervention) |
| Becker et al. [12] | Inpatient and outpatient, different diagnoses | No control group | KEN-Online, transdiagnostic psychodynamic online self-help program | Principles of mindfulness and affect phobia | Enhancing awareness of own emotions, regulating emerging anxiety, fully experiencing emotions, expressing emotions to other people | Significant reduction of anxiety and depression (t2 to last T-weekly measurements), reported higher emotional competence, acceptance not measured | 7.6% of participants completed all units |
| Richter et al. [13] | Inpatient, depression | TAU *(not specified)* | Deprexis | Self-help | 12 week program with modules | Significant improvement regarding subjective depression-severity and daily activity | *Not reported* |
| Dorow et al. [14] | Inpatient, depression | No control group | Moodgym, a CBT-based self-management app | CBT self-help | Interactive CBT skill training focused on feelings, thoughts, cognitive restructuring, stress management, and relationships | Moderate to high acceptance according to USE score | Low to moderate adherence (used by 59%) |
| Herpertz et al. [15] | Inpatient, Schizophrenia | No control group | Digital assessment of longitudinal symptoms via online questionnaires | None | Symptom assessment via self-report questionnaires | 98% reported high satisfaction | *Not reported* |
| Greenwood et al. [16] | Inpatient, veterans with substance abuse | No control group, qualitative interviews | SlowMo | CBT-based cognitive restructuring of paranoid beliefs | 11 sessions of talking therapy focused on worries and thinking habits | Importance of therapist relationship, guidance, and social support; theoretical framework is helpful; some challenges regarding technology | *Not reported* |
| Bass et al. [17] | Inpatient, veterans | No control group (survey) | Various eHealth tools used in the context of inpatient veteran treatment | NA | NA | 20% use eHealth technology, mostly tablets and video chats; usage is desired but low. | *Not reported* |
| Gupta et al. [18] | Inpatient, various | No control group (qualitative study) | Minddistrict | Transdiagnostic self-help modules | Modules include psychoeducation, exposure training, and behaviour tracking through diaries. | Acceptance and satisfaction higher for patients than for staff; limitations are work load, insufficient time, lack of appropriate setting | *Not reported* |
| Riches et al. [19] | Inpatient and outpatient, various | No control group | VR virtual relaxation | Stress management, CBT | Immersion into a calming VR environment, guided by therapists with CBT-based techniques | VR perceived as enjoyable, relaxing and helpful; number of violent incidents halved | *Not reported* |
| Wälchli et al. [20] | Inpatient, various | RCT TAU | REMOTION + TAU | Web-based emotion regulation intervention; external process model | Transdiagnotic treatment focusing on identification, selection and implementation of emotions consisting of 6 modules | Good usability and satisfaction; no impact on symptom severity or emotion regulation | Low adherence (12 out of 30 participants completed at least 50%) |
| Bielinski et al. [21] | Inpatient, various | No control (qualitative interviews) | REMOTION | Web-based emotion regulation intervention; external process model | Transdiagnotic treatment focusing on identification, selection and implementation of emotions consisting of 6 modules | Themes: lack of previous experience, intervention as a complement, concerns on fit for acute psychiatric care, importance of the human factor, further requirements such as resources and time | *Not reported* |

**References**

1. Perlich A, Meinel C. Patient-Provider Teamwork via Cooperative Note Taking on Tele-Board MED. Stud Health Technol Inform. 2016;228:117-21.
2. Zwerenz R, Becker J, Knickenberg RJ, Siepmann M, Hagen K, Beutel ME. Online Self-Help as an Add-On to Inpatient Psychotherapy: Efficacy of a New Blended Treatment Approach. Psychother Psychosom. 2017;86(6):341-50.
3. Zwerenz R, Baumgarten C, Becker J, Tibubos A, Siepmann M, Knickenberg RJ, et al. Improving the Course of Depressive Symptoms After Inpatient Psychotherapy Using Adjunct Web-Based Self-Help: Follow-Up Results of a Randomized Controlled Trial. J Med Internet Res. 2019;21(10):e13655.
4. Hammond AS, Antoine DG, Stitzer ML, Strain EC. A Randomized and Controlled Acceptability Trial of an Internet-based Therapy among Inpatients with Co-occurring Substance Use and Other Psychiatric Disorders. J Dual Diagn. 2020;16(4):447-54.
5. Bendig E, Küchler AM, Baumeister H, Becker T. Blended Care in In-Patient Acute Psychiatric Care. The Example of a Group Training for Social Competences in Adults-A Pretest-Posttest Feasibility Study. Int J Environ Res Public Health. 2021;18(18).
6. Nolte S, Busija L, Berger T, Meyer B, Moritz S, Rose M, et al. Do sociodemographic variables moderate effects of an internet intervention for mild to moderate depressive symptoms? An exploratory analysis of a randomised controlled trial (EVIDENT) including 1013 participants. BMJ Open. 2021;11(1):e041389.
7. Kreis A, Becker J, Beutel ME, Zwerenz R. Einfluss akzeptanzfördernder Interventionen auf die Nutzung einer Online-Selbsthilfe. Psychotherapeut. 2021;66(5):406-13.
8. Sharma G, Schlosser L, Jones BDM, Blumberger DM, Gratzer D, Husain MO, et al. Brief App-Based Cognitive Behavioral Therapy for Anxiety Symptoms in Psychiatric Inpatients: Feasibility Randomized Controlled Trial. JMIR Form Res. 2022;6(11):e38460.
9. Schwarz J, Mauche N, Oehler C, Rummel-Kluge C, Hegerl U, Strauss M. ["iFightDepression" in inpatient treatment : Evaluation of a web-based, therapist-guided self-management program on a specialized ward for affective disorders]. Nervenarzt. 2022;93(5):459-67.
10. Van Assche E, Bonroy B, Mertens M, Van den Broeck L, Desie K, Bolinski F, et al. E-mental health implementation in inpatient care: Exploring its potential and future challenges. Front Digit Health. 2022;4:1027864.
11. Levis M, Ludmer DJ, Cornelius S, Scott R, Watts BV, Shiner B. An implementation and effectiveness study evaluating Conflict Analysis in VA residential substance abuse services: Whole Health informed self-guided online care. Explore (NY). 2022;18(6):688-97.
12. Becker J, Kreis A, Schorch T, Mayer A, Tsiouris A, Beutel ME, et al. Adherence and effectiveness of an emotion-based psychodynamic online self-help during and after inpatient and day-care psychotherapy: Results of a naturalistic study. Front Psychiatry. 2023;14:1027118.
13. Richter LE, Machleit-Ebner A, Scherbaum N, Bonnet U. How Effective is a Web-Based Mental Health Intervention (Deprexis) in the Treatment of Moderate and Major Depressive Disorders when started during Routine Psychiatric Inpatient Treatment as an Adjunct Therapy? A Pragmatic Parallel-Group Randomized Controlled Trial. Fortschr Neurol Psychiatr. 2023;91(7-08):297-310.
14. Dorow M, Stein J, Förster F, Löbner M, Franz M, Günther R, Schröder R, Sommer D, Möller D, Dekoj MC, Becker T. Der komplementäre Einsatz des internetbasierten Selbstmanagementprogramms „moodgym “bei Menschen mit depressiven Erkrankungen in der stationären Versorgung–die Perspektive von Patienten und Behandlern. Psychiatrische Praxis. 2018 Jul;45(05):256-62.
15. Herpertz J, Richter MF, Barkhau C, Storck M, Blitz R, Steinmann LA, et al. Symptom monitoring based on digital data collection during inpatient treatment of schizophrenia spectrum disorders - A feasibility study. Psychiatry Res. 2022;316:114773.
16. Greenwood KE, Gurnani M, Ward T, Vogel E, Vella C, McGourty A, et al. The service user experience of SlowMo therapy: A co-produced thematic analysis of service users' subjective experience. Psychol Psychother. 2022;95(3):680-700.
17. Bass E, Garabrant J, Salyers MP, Patterson S, Iwamasa GY, McGuire AB. eHealth Use on Acute Inpatient Mental Health Units: Implementation Processes, Common Practices, and Barriers to Use. Adm Policy Ment Health. 2023;50(4):603-15.
18. Gupta N, Leuba S, Seifritz E, Berger T, Kawohl W. Resources, support, and integration as potential barriers and facilitators to the implementation of blended therapy in the routine care of inpatients: a qualitative study. Frontiers in Psychiatry. 2024 Dec 17;15:1417784.
19. Riches S, Nicholson SL, Fialho C, Little J, Ahmed L, McIntosh H, Kaleva I, Sandford T, Cockburn R, Odoi C, Azevedo L. Integrating a virtual reality relaxation clinic within acute psychiatric services: a pilot study. Psychiatry research. 2023 Nov 1;329:115477.
20. Wälchli G, Berger T, Nissen C, Moggi F, Bielinski LL. Examining the potential of an internet-based emotion regulation intervention added to acute psychiatric inpatient care: results from a randomized controlled pilot trial. Psychiatry Research. 2025 Feb 1;344:116326.
21. Bielinski LL, Wälchli G, Lange A, von Känel E, Demel LK, Nissen C, Moggi F, Berger T. A qualitative analysis of healthcare professionals’ experiences with an internet-based emotion regulation intervention added to acute psychiatric inpatient care. BMC psychiatry. 2024 Dec 27;24(1):955.
