# Supplementary material for: E-Mental Health Interventions in Inpatient Care: Scoping Review
Source: J Med Internet Res. 2025 Jul 31;27:e65947. doi: 10.2196/65947 (PMC12355140; doi:10.2196/65947)
Supplement: Multimedia Appendix 4 [file jmir_v27i1e65947_app4.docx]

**Multimedia Appendix 4.** Summary of e-mental health aftercare studies included in this scoping review.

| **Author (year)** | **Patient condition** | **Control group** | **EMH type** | **Type of therapy** | **Content** | **Results and Acceptance** | **Adherence** |
| --- | --- | --- | --- | --- | --- | --- | --- |
| Schmädeke & Bischoff [1] | Depression | Treatment as usual (TAU) | Mobile app | cognitive-behavioural rehabilitation aftercare | Self-regulation, Planning of daily structure, relaxation exercises, contact with online coach | Significant improvement of depressive symptoms, significant effect regarding self-motivation and self-efficacy  Acceptance deemed as good (no measurement) | 100% complete cases |
| Kordy et al. [2] | Depression | TAU | Mobile app | maintenance cognitive-behavioural therapy (CBT) | Monitoring: signalling upcoming crises, assistance with personal crisis management and facilitation of early intervention | Intervention reduced time with “unwell” status, facilitated faster transition from unwell to well, no significant results  Acceptance deemed as good (no measurement) | 97% completer |
| Zwerenz et al. [3] | Different diagnoses | access to placebo Internet-based program containing links to publicly accessible information about stress management and coping | Web-based | manualized, educational vocational training | writing of personally emotionally meaningful experiences with a focus on workplace related experiences | overall well accepted and improvements in IG compared to CG for a variety of chronic mental and somatic conditions, although with small effect sizes;  acceptance deemed as good (no measurement) | 65% used intervention |
| Jacobi et al. [4] | Bulimia nervosa | TAU | Web-based | CBT | interactive features, individualized email feedback, real-time individual chats, web-based sessions | no significant difference  Acceptance not measured | 36% opened program |
| Zwerenz et al. [5] | Different diagnoses | Wait-list control group | Web-based | Psychodynamic self-help | guided version of self-help-online-intervention | Significant improvement of depressive symptoms, quality of life and emotional competence  Acceptance high, 78% rated program as good or very good | 36% completed more than 50% of sessions |
| Kraft et al. [6] | Depressive symptoms | access to mindfulness training group | Mobile text-messages | mindfulness | Mindfulness home practice through text messaging | Feasible for some but not all patients  Acceptance deemed as good (no measurement) | 81% used intervention at least once |
| Välimäki et al. [7] | Psychosis | TAU | Mobile text-messages | self-determination theory | text messages reminding patients of treatment-related appointments and activities during free time | No significant improvements  Acceptance good, 72% satisfied | 95.2% completers |
| Linden & Pirsich [8] | Different diagnoses | No control group | Internet chat forum | Self-help group | Chat forum with or without moderation | No measurement | 8% of patients participated, 32% and 43% of participants used the intervention |
| Lange et al. [9] | Anxiety symptoms, depression symptoms because of somatic illness | TAU | Internet chat group | Behaviour-therapy-oriented | Guided online chat group | Poorer results in intervention group  Acceptance deemed as good (78% of participants would recommend the intervention, self-developed questionnaire) | 33% attended all five offered sessions |
| Schlicker et al. [10] | Major depressive disorder | Wait-list control group | Mobile text-messages | Emotion regulation skills | standardized messages targeting emotion regulation | significantly smaller increase of depressive symptoms during post-treatment and follow-up interval | 40% in intervention group completed |
| Norlund et al. [11] | Anxiety symptoms, depression symptoms because of somatic illness | TAU | Therapist guided online CBT + modules with information and trainings | CBT | 14-week, therapist-guided intervention with 10 different modules consisting of self-monitoring, skills training, and engagement in exercises based on CBT techniques | No significant results | 15.4% completed additional modules, only 0.9% completed recommended modules of program |
| Beutel et al. [12] | Depressive symptoms | Active control group (access to placebo Internet-based program containing links to publicly accessible information about depression) | Deprexis | CBT self-help | Interactive web-based self-help program | Significant improvement regarding depression scores, anxiety, quality of life, self-esteem | *No data reported* |
| Zwerenz et al. [13] | Rehabilitants with Work-related stress | Active control group | Web-based work-related psychological aftercare program (GSA) | Structured psychodynamic | Weekly personalized writing impulses from therapist, additional therapeutic commentaries to the diary entries | Generally high acceptance, 43.5% largely and 45.7% very satisfied. Significant improvement observed regarding subjective work ability | 58.1% completed at least 6 diary entries |
| Neumayr et al. [14] | Anorexia nervosa | TAU (information about post discharge treatment) | Smartphone-based aftercare intervention | CBT with additional elements of DBT, ACT and MET | Therapist feedback additional to self-monitoring, positive reinforcement, meal planner and others | Good acceptability, 29.4% app very helpful, 53.9% helpful and 17.6% moderately helpful. Patients reported positive effect of app | Only one patient discontinued the app (5%), patients used app an average of 4 times per day |
| Gallinat et al. [15] | Schizophrenia | TAU | “Heidelberg Internet-based aftercare for patients with schizophrenia spectrum disorders” (HEINS) | Supportive monitoring focus | Modules used as add-on to conventional care for six months following inpatient treatment, supportive monitoring by therapist as central module | All participants reported program as appropriate aftercare, 9/10 would participate again. Study not designed to assess treatment benefits | 70.7% of total monitoring assessments were completed by participants |
| Becker et al. [16] | Rehabilitants with Work-related stress | Active control group, access to health-beneficial online information | Web-based work-related psychological aftercare program (GSA) | Supportive-expressive-therapy (SET) after L. Luborsky (1995) | Twelve week web-based intervention with self-written blog posts and therapeutic comments | Significant reduction in anxiety, depressive symptoms and stress | 91% logged in at least once |
| Bruhns et al. [17] | Depression | Wait-list control group | Mobile-based aftercare intervention “COGITO” | Metacognitive (group) training, CBT and third wave approaches | Short exercises on topics of cognitive strategies, communication and interaction, positive activities, mindfulness and imagination, metacognitive training | Significant increase in self-competence. Self-esteem, quality of life and depressive symptoms were not significantly improved. Acceptance not reported | Drop-out rate of 23.3% |
| Richter et al. [18] | Depression | TAU | Deprexis | CBT self-help | Interactive web-based self-help program | Improved depression severity and daily activity; more post-inpatient stability | *Not reported* |
| Cardi et al. [19] | Anorexia | TAU | ECHOMANTRA | Self-management | Workbooks, videos, and online groups | No superiority to TAU, good acceptance | Only 20% met adherence requirements |
| Lauvsnes et al. [20] | Substance use disorder | No control group | EMA | Not applicable | Neurophysiological assessment via mobile devices | Craving intensity identified as the most important predictor of substance use episodes | *Not reported* |
| Cardi et al. [21] | Anorexia | No control group (qualitative interviews) | ECHOMANTRA | Self-management | Workbooks, videos, and online groups | Materials were found useful and easy to access; obstacles was lack of time | Only 20% met adherence |
| Gaudiano et al. [22] | Psychosis | No control group | Multiple mobile app interventions | CBT for psychosis | Psychoeducation, mobile after-care support | Good acceptance | high engagement |
| Clark Bryan et al. [23] | Anorexia | No control group (qualitative interviews) | ECHOMANTRA | Self-management | Workbooks, videos, and online groups | Five themes: mixed experience; tailoring to stage of recovery; carer involvement; acceptability of remote support; impact of self-monitoring and accountability | *Not reported* |
| Suzuki et al. [24] | Alcohol use disorder | No control group | Brief Addiction Monitor | Peer recovery coach | Contact with coach for daily motivation, appointment reminders, and inquiry about alcohol use and risk and protective factor | Increased linking with outpatient treatment for patients who engaged with the coach | Moderate adherence |

**References**

1. Schmädeke S, Bischoff C. Wirkungen smartphonegestützter psychosomatischer Rehabilitationsnachsorge (eATROS) bei depressiven Patienten. Verhaltenstherapie. 2015;25(4):277-86.
2. Kordy H, Wolf M, Aulich K, Bürgy M, Hegerl U, Hüsing J, et al. Internet-Delivered Disease Management for Recurrent Depression: A Multicenter Randomized Controlled Trial. Psychother Psychosom. 2016;85(2):91-8.
3. Zwerenz R, Becker J, Gerzymisch K, Siepmann M, Holme M, Kiwus U, et al. Evaluation of a transdiagnostic psychodynamic online intervention to support return to work: A randomized controlled trial. PLoS One. 2017;12(5):e0176513.
4. Jacobi C, Beintner I, Fittig E, Trockel M, Braks K, Schade-Brittinger C, et al. Web-Based Aftercare for Women With Bulimia Nervosa Following Inpatient Treatment: Randomized Controlled Efficacy Trial. J Med Internet Res. 2017;19(9):e321.
5. Zwerenz R, Becker J, Johansson R, Frederick RJ, Andersson G, Beutel ME. Transdiagnostic, Psychodynamic Web-Based Self-Help Intervention Following Inpatient Psychotherapy: Results of a Feasibility Study and Randomized Controlled Trial. JMIR Ment Health. 2017;4(4):e41.
6. Kraft S, Wolf M, Klein T, Becker T, Bauer S, Puschner B. Text Message Feedback to Support Mindfulness Practice in People With Depressive Symptoms: A Pilot Randomized Controlled Trial. JMIR Mhealth Uhealth. 2017;5(5):e59.
7. Välimaki M, Kannisto KA, Vahlberg T, Hatonen H, Adams CE. Short Text Messages to Encourage Adherence to Medication and Follow-up for People With Psychosis (Mobile.Net): Randomized Controlled Trial in Finland. J Med Internet Res. 2017;19(7):e245.
8. Linden M, Pirsich C. Negative and helpful statements in a patient only or therapist guided internet forum in the aftercare for psychosomatic inpatients. Psychiatr Danub. 2017;29(4):446-50.
9. Lange L, Fink J, Bleich C, Graefen M, Schulz H. Effectiveness, acceptance and satisfaction of guided chat groups in psychosocial aftercare for outpatients with prostate cancer after prostatectomy. Internet Interv. 2017;9:57-64.
10. Schlicker S, Ebert DD, Middendorf T, Titzler I, Berking M. Evaluation of a text-message-based maintenance intervention for Major Depressive Disorder after inpatient cognitive behavioral therapy. J Affect Disord. 2018;227:305-12.
11. Norlund F, Wallin E, Olsson EMG, Wallert J, Burell G, von Essen L, et al. Internet-Based Cognitive Behavioral Therapy for Symptoms of Depression and Anxiety Among Patients With a Recent Myocardial Infarction: The U-CARE Heart Randomized Controlled Trial. J Med Internet Res. 2018;20(3):e88.
12. Beutel ME, Becker J, Hagen K, Siepmann M, Knickenberg RJ, Zwerenz R. Does blended online care improve outcomes of inpatient psychotherapy? An RCT with an additional online self-help program. Journal of Psychosomatic Research. 2018;109:91-2.
13. Zwerenz R, Baumgarten C, Dahn I, Labitzke N, Schwarting A, Rudolph M, et al. Implementation of a Web-Based Work-Related Psychological Aftercare Program Into Clinical Routine: Results of a Longitudinal Observational Study. J Med Internet Res. 2019;21(6):e12285.
14. Neumayr C, Voderholzer U, Tregarthen J, Schlegl S. Improving aftercare with technology for anorexia nervosa after intensive inpatient treatment: A pilot randomized controlled trial with a therapist-guided smartphone app. Int J Eat Disord. 2019;52(10):1191-201.
15. Gallinat C, Moessner M, Apondo S, Thomann PA, Herpertz SC, Bauer S. Feasibility of an Intervention Delivered via Mobile Phone and Internet to Improve the Continuity of Care in Schizophrenia: A Randomized Controlled Pilot Study. Int J Environ Res Public Health. 2021;18(23).
16. Becker J, Kreis A, Beutel ME, Zwerenz R. [Effectiveness of the Internet-Based, Job-Related Aftercare GSA-Online Following Inpatient Psychosomatic Rehabilitation: Results of a Randomized Controlled Trial]. Rehabilitation (Stuttg). 2022;61(4):276-86.
17. Bruhns A, Baumeister A, Demeroutis G, Jahn H, Willenborg B, Shaffy A, et al. A mobile-based aftercare intervention to increase self-esteem in inpatients diagnosed with depression: A randomized controlled trial. Psychother Res. 2023;33(6):783-802.
18. Richter LE, Machleit-Ebner A, Scherbaum N, Bonnet U. How effective is a web-based mental health intervention (Deprexis) in the treatment of moderate and major depressive disorders when started during routine psychiatric inpatient treatment as an adjunct therapy? A pragmatic parallel-group randomized controlled trial. Fortschritte der Neurologie· Psychiatrie. 2023 Jul;91(07/08):297-310.
19. Cardi V, Rowlands K, Ambwani S, Macdonald P, Arcelus J, Schmidt U, Treasure J. Patient and carer feedback and engagement with ECHOMANTRA, a digital guided intervention for anorexia nervosa. European Eating Disorders Review. 2025 Mar;33(2):210-20.
20. Lauvsnes AD, Hansen TI, Ankill SØ, Bae SW, Gråwe RW, Braund TA, Larsen M, Langaas M. Mobile assessments of mood, cognition, smartphone-based sensor activity, and variability in craving and substance use in patients with substance use disorders in Norway: prospective observational feasibility study. JMIR Formative Research. 2023 Jun 23;7:e45254.
21. Cardi V, Rowlands K, Ambwani S, Lord J, Clark-Bryan D, McDaid D, Schmidt U, Macdonald P, Arcelus J, Landau S, Treasure J. Efficacy and cost-effectiveness of a digital guided self-management intervention to support transition from intensive care to community care in anorexia nervosa (TRIANGLE): pragmatic multicentre randomised controlled trial and economic evaluation. EClinicalMedicine. 2024 Jul 1;73.
22. Gaudiano BA, Ward M, Benz MB, Hughes C, Johnson JE, Moitra E. Feasibility and acceptability of a mobile intervention for patients with psychosis following psychiatric hospitalization: A pilot randomized controlled trial. Psychological Services. 2024 Jun 6.
23. Bryan DC, Rowlands K, Macdonald P, Cardi V, Ambwani S, Arcelus J, Landau S, Schmidt U, Treasure J. Transition support for patients admitted to intensive treatment for anorexia nervosa: qualitative study of patient and carer experiences of a hybrid online guided self-help intervention (ECHOMANTRA). BJPsych Open. 2024 May;10(3):e81.
24. Suzuki J, Loguidice F, Prostko S, Szpak V, Sharma S, Vercollone L, Garner C, Ahern D. Digitally assisted peer recovery coach to facilitate linkage to outpatient treatment following inpatient alcohol withdrawal treatment: Proof-of-concept pilot study. JMIR formative research. 2023 Jul 5;7(1):e43304.
